# The optimal diagnostic criteria of endogenous hyperinsulinemic hypoglycemia based on a large cohort of Chinese patients

**DOI:** 10.3389/fendo.2022.994707

**Published:** 2022-10-19

**Authors:** Jie Yu, Yiwen Liu, Lu Lyu, Yuan Zhao, Mengya Qi, Fan Ping, Lingling Xu, Wei Li, Qiang Xu, Huabing Zhang, Wenming Wu, Yuxiu Li

**Affiliations:** ^1^Key Laboratory of Endocrinology of National Health Commission, Department of Endocrinology, Peking Union Medical College Hospital, Chinese Academy of Medical Sciences & Peking Union Medical College, Beijing, China; ^2^Department of General Surgery, Peking Union Medical College Hospital, Chinese Academy of Medical Sciences & Peking Union Medical College, Beijing, China

**Keywords:** C-peptide, hypoglycemia, insulinoma, insulin, proinsulin

## Abstract

**Purpose:**

An end-of-fast insulin level ≥ 3 µIU/ml, C-peptide level ≥ 0.6 ng/ml, and proinsulin level ≥ 5 pmol/l with end-of-fast glucose level ≤ 3.0 mmol/l have been established as the criteria for endogenous hyperinsulinemic hypoglycemia. However, all these criteria have been proposed based on patients in Western populations. This study aimed to determine the optimal criteria using a large series of Chinese patients.

**Methods:**

This retrospective study comprised 144 patients with surgically proven insulinoma and 40 controls who underwent a 72-h fasting test at the Peking Union Medical College Hospital(PUMCH) from 2000 to 2020. Receiver operating characteristic curves were used for analysis.

**Results:**

In this series of patients, the optimal diagnostic criteria for endogenous hyperinsulinemic hypoglycemia were insulin  ≥ 5.5 μIU/ml, C-peptide ≥ 0.7 ng/ml, and proinsulin ≥ 12 pmol/l with end-of-fast glucose ≤ 2.8 mmol/l; the sensitivity and specificity were 99% and 100% for insulin, 100% and 100% for C-peptide, and 93% and 100% for proinsulin, respectively. The diagnostic efficacy of the criteria based on Western populations was then tested. The sensitivity and specificity of end-of-fast insulin ≥ 3 μIU/ml, C-peptide ≥ 0.6 ng/ml, and proinsulin ≥ 5 pmol/l with end-of-fast glucose ≤ 3.0 mmol/l were 100% and 83%, 100% and 80%, and 97% and 78%, respectively.

**Conclusions:**

New and optimized diagnostic criteria for endogenous hyperinsulinemic hypoglycemia in Chinese populations have been proposed, and these criteria yield satisfactory accuracy.

## Introduction

Insulinoma is a rare pancreatic neuroendocrine tumor with a prevalence of approximately 1 to 4 cases per 1 million people (1, 2). Subjects with insulinoma experience hypoglycemia with Whipple’s triad in the fasting state (3). The prolonged 72-h fasting test is the classic diagnostic test for hypoglycemia (4, 5). For healthy individuals, when the fasting blood glucose level decreases to ≤ 2.8 mmol/l, the secretion of β-cell polypeptide is suppressed, but this is not the case for patients with insulinoma (3, 4). Thus, hypoglycemia in patients with insulinoma is caused by inappropriate endogenous hyperinsulinism (3).

The most widely used biochemical diagnostic criteria of endogenous hyperinsulinemic hypoglycemia are as follows: insulin level ≥ 3 μIU/l, C-peptide level ≥ 0.6 ng/ml, and proinsulin level ≥ 5 pmol/l with end-of-fast glucose ≤ 3.0 mmol/l (4, 6–8). These criteria were recommended in practice guidelines for the diagnosis of hypoglycemic disorders by the Endocrine Society in 2009 (9). However, these criteria, which were proposed mainly by Professor Service from Mayo Clinic and were based on Western populations, have never been tested in Asian populations. Moreover, up to now, there have been no studies conducted in Asian populations reporting the criteria for endogenous hyperinsulinemic hypoglycemia.

Therefore, the present study aimed to determine the best parameters and threshold levels in the diagnosis of endogenous hyperinsulinemic hypoglycemia using a relatively large cohort of insulinoma patients examined at the Peking Union Medical College Hospital (PUMCH) in China from 2000 to 2020. In addition, the study evaluated the diagnostic performance of the established standards from Western populations in our patient cohort.

## Subjects, materials, and methods

### Study population

Data were retrospectively acquired from the PUMCH medical record system from 2000 to 2020. Consecutive patients diagnosed and admitted at the PUMCH and surgically diagnosed with insulinoma were included. Control subjects were those who were referred to undergo assessments related to hypoglycemia but considered healthy after successful completion of the 72-h fasting test along with no finding of insulinoma during follow-up. In total, 144 patients with insulinoma from 2012 to 2020 and 40 control subjects from 2000 to 2020 were included in this study.

The prolonged fasting test was conducted according to a standard protocol on patients admitted to the PUMCH. The fast was terminated when both the plasma glucose level was ≤ 2.8 mmol/l and the patient had symptoms and signs of hypoglycemia. For subjects who failed to develop biochemical and symptomatic hypoglycemia, the fast was terminated at 72 h.

### Clinical assays

The glucose oxidase assay was performed to determine the serum glucose level. The serum insulin and C-peptide levels were examined using chemiluminescence assays (ADVIA Centaur XP, Siemens). The detection ranges for the insulin and C-peptide assays were 1–300 μIU/ml and 0.05–30 ng/ml, respectively. For proinsulin, only data after April 2015 were analyzed, and a chemiluminescence immunoassay (MAGLUMI 2000, Snibe, Co., Ltd.) was used. The detection range for the proinsulin assay was 0.2–500 pmol/l. All tests were performed in the clinical laboratory of the PUMCH.

This study was approved by the Institutional Review Board of PUMCH, Chinese Academy of Medical Sciences.

### Statistical analyses

Continuous data were expressed as the mean ± standard deviations when they were normally distributed or as the median (interquartile range) when they were non-normally distributed. Descriptive data for categorical variables were summarized using frequencies or percentages. A Student t-test was performed to compare the means of the continuous data with a normal distribution, whereas the Mann–Whitney U test was used for continuous data with a non-normal distribution. The chi-squared test was used to compare categorical variables. The receiver operating characteristic (ROC) curves were generated, and the areas under the ROC curves (AUC-ROCs) were calculated to evaluate the discriminatory power of insulin, C-peptide, and proinsulin to distinguish between patients with insulinoma and controls. In ROC analysis, the optimal cutoff value maximized the Youden index, i.e., sensitivity + specificity – 1 (10). Statistical analyses were performed using International Business Machines Statistical Package for the Social Sciences Statistics version 25.0 (IBM Corp., Armonk, NY, USA). A two-tailed P < 0.05 was considered significant.

## Results

### Blood glucose, insulin, C-peptide, and proinsulin at discontinuation of the prolonged 72-h fasting test

The patients with insulinoma had lower average blood glucose levels, as suggested by lower hemoglobin A1c and glycated albumin (%), than controls (**Table 1**). At discontinuation of the prolonged 72-h fasting test, patients with insulinoma had lower blood glucose levels and significantly higher proinsulin, insulin, and C-peptide levels than controls (**Table 1**). Anti-insulin antibody titers were below the limit of quantification in both groups (**Table 1**).

**Table 1:** Basic characteristics of patients with insulinoma and controls.

Characteristics	Insulinoma (n = 144)	Controls (n = 40)	P value
Female, n (%)	103 (62.0)	26 (49.1)	0.094
Age (years)	45.9 ± 13.6	43.7 ± 12.6	0.309
Height (cm)	163.7 ± 9.0	165.9 ± 8.1	0.115
Weight (kg)	73.1 ± 14.7	69.4 ± 14.1	0.106
BMI (kg/m^2^)	27.2 ± 4.8	25.1 ± 4.2	**0.004**
WC (cm)	93.6 ± 11.5	90.3 ± 13.0	0.170
HbA1c (%)	4.7 ± 0.4	5.3 ± 0.5	**< 0.001**
GA (%)	10.7 ± 1.7	13.1 ± 1.2	**< 0.001**
IAA titers (IU/mL)	0	0	**-**
Tumor diameter(mm)	15(9.5)	–	**-**
Results of prolonged fasts			
Glucose (mmol/l)	2.2 ± 0.5	3.4 ± 0.7	**< 0.001**
Insulin (μIU/ml)	23.31 (33.3)	2.67 (2.85)	**< 0.001**
C-peptide (ng/ml)	2.86 (2.22)	0.44 (0.39)	**< 0.001**
Proinsulin (pmol/l)	85.6 (186.5)	4.2 (5.9)	**< 0.001**

BMI, body mass index; WC, waist circumference; HbA1c, hemoglobin A1c; GA, glycated albumin; IAA, insulin autoantibody.

Continuous data were expressed as the mean ± standard deviations when normally distributed or as the median (interquartile range) when non-normally distributed. P < 0.05 was considered significant and was presented in bold.

At the end of the fast, blood glucose concentrations in insulinoma patients overlapped with those of the control group, but the distribution pattern was different (**Supplementary Figure 1**, **Supplementary Table 1**).

We further conducted Spearman correlation analysis and found that in the control group, blood glucose level was significantly positively correlated with insulin, C-peptide, and proinsulin levels, whereas in the insulinoma group, blood glucose level was significantly negatively correlated with C-peptide and insulin levels (**Supplementary Table 2**). The negative correlation between proinsulin and blood glucose levels in the insulinoma group did not reach significance (**Supplementary Table 2**).

### New and optimal diagnostic criteria for endogenous hyperinsulinemic hypoglycemia based on our cohort

Because β-cell polypeptide secretion is associated with blood glucose, especially in healthy individuals, the diagnostic performance of insulin, C-peptide, and proinsulin can be different with different concomitant glucose levels (11, 12). Thus, we explored the diagnostic criteria and their diagnostic accuracy at different end-of-fast blood glucose concentrations, such as ≤ 3.3, ≤ 3.0, and ≤ 2.8 mmol/. In addition, we further evaluated the diagnostic criteria and accuracy when the blood glucose level was between 2.8 and 3.3 mmol/l, with often ambiguous and difficult to interpret results. The median (range) levels of insulin, C-peptide, and proinsulin by different glucose levels are shown in **Supplementary Table 3**.

**Figure 1:**
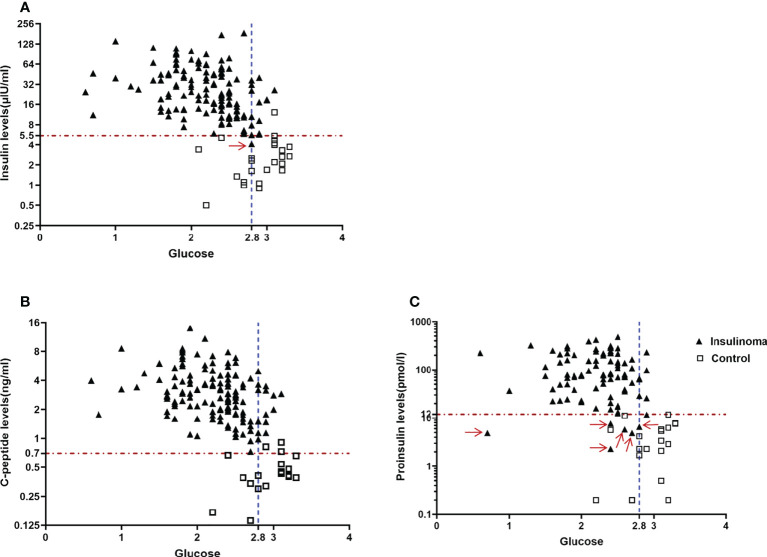
New and optimal diagnostic criteria for endogenous hyperinsulinemic hypoglycemia based on our cohort. **(A)** An end-of-fast insulin level>=5.5μIU/ml with concomitant blood glucose level<=2.8mmol/L missed one patient with insulinoma(Red arrow), **(B)** An end-of-fast C-peptide level>=0.7ng/ml with concomitant blood glucose level<=2.8mmol/L completely distinguished the patients with insulinoma from the control group, and **(C)** An end-of-fast proinsulin >=12pmol/L with concomitant blood glucose level<=2.8mmol/L missed 6 patients with insulinoma (Red arrow).

Newly established criteria of insulin, C-peptide, and proinsulin for the diagnosis of endogenous hyperinsulinemic hypoglycemia were derived by ROC analysis in our cohort. We found the best criteria were insulin ≥ 5.5 μIU/ml, C-peptide ≥ 0.7 ng/ml, and proinsulin ≥ 12 pmol/l with end-of-fast glucose ≤ 2.8 mmol/l. The sensitivity and specificity were 99% and 100% for insulin, 100% and 100% for C-peptide, and 93% and 100% for proinsulin, respectively (**Table 2**, **Figure 1**).

**Table 2:** Diagnostic performance of new criteria in our study and previous criteria based on Western populations with concomitant blood glucose below 2.8 or 3.0mmol/L.

	Patients Meeting Criteria		Sensitivity %(95% CI)	Specificity %(95% CI)	PPV %(95% CI)	NPV %(95% CI)
	Insulinoma (n/n)	Control (n/n)				
**This study’s criteria**						
Glucose<=3.0mmol/L						
insulin>=5.5μIU/ml	141/142	0/12	99.3 (95.6,99.9)	100 (69.9,100)	100 (96.7,100)	92.3 (62.1,99.6)
C-peptide>=0.9ng/ml	139/140	0/10	99.3 (95.5,99.9)	100 (65.5,100)	100 (96.7,100)	90.9 (57.1,99.5)
Proinsulin>=12pmol/L	86/93	0/9	92.5 (84.6,96.7)	100 (62.9,100)	100 (94.7,100)	56.3 (30.6,79.2)
Glucose<=2.8mmol/L						
insulin>=5.5μIU/ml	134/135	0/9	99.3 (95.3,100)	100 (62.9,100)	100 (96.5,100)	90 (54.1,99.5)
C-peptide>=0.7ng/ml	133/133	0/8	100 (96.5,100)	100 (59.8,100)	100 (96.5,100)	100 (59.8,100)
Proinsulin>=12pmol/L	83/89	0/8	93.2 (85.4,97.2)	100 (59.8,100)	100 (94.5,100)	57.1 (29.6,81.2)
**Previous criteria**						
Glucose<=3.0mmol/L						
insulin>=3.0μIU/ml	142/142	2/12	100 (96.7,100)	83.3 (50.9,97.1)	98.6 (94.5,99.8)	100 (65.5,100)
C-peptide>=0.6ng/ml	140/140	2/10	100 (96.7,100)	80 (44.2,96.5)	98.6 (94.5,99.8)	100 (59.8,100)
Proinsulin>=5pmol/L	90/93	2/9	96.9 (90.2,99.2)	77.8 (40.2,96.1)	97.8 (91.6,99.6)	70 (35.4,91.9)
Glucose<=2.8mmol/L						
insulin>=3.0μIU/ml	135/135	2/9	100 (87.7,100)	77.8 (40.2,96.1)	94.6 (94.6,99.1)	100 (56.1,100)
C-peptide>=0.6ng/ml	132/133	1/8	99.2 (95.3,99.9)	87.5 (46.7,99.3)	99.2 (95.3,99.9)	87.5 (46.7,99.3)
Proinsulin>=5pmol/L	86/89	2/8	96.6 (89.7,99.1)	75 (35.6,95.5)	97.7 (91.3,99.6)	66.7 (30.9,90.9)

CI, confidence interval; PPV, positive predictive value; NPV, negative predictive value.

When the upper limit of the concomitant blood glucose level increased from 2.8 to 3.3 mmol/l, the specificity of insulin and C-peptide slightly decreased. If the concomitant blood glucose level was between 2.8 and 3.3 mmol/L, insulin ≥ 5.5 μIU/ml, C-peptide ≥ 0.9 ng/ml, and proinsulin ≥ 8 pmol/l all reached 100% sensitivity but had relatively poor specificity. However, if we did not consider the concomitant blood glucose level, these parameters achieved the poorest diagnostic accuracy (**Supplementary Table 4**).

### Diagnostic performance of western population-based criteria for endogenous hyperinsulinemic hypoglycemia in our cohort

Using the most widely used cutoff values, 142 in 142, 140 in 140, and 90 in 93 of patients with insulinoma had end-of-fast insulin, C-peptide, and proinsulin concentrations of ≥ 3 μIU/ml (sensitivity, 100%), ≥ 0.6 ng/ml (sensitivity, 100%), and ≥ 5 pmol/l (sensitivity, 97%), respectively. However, only 10 in 12, 8 in 10, and 7 in 9 of control patients had end-of-fast insulin, C-peptide, and proinsulin concentrations of < 3 μIU/ml (specificity, 83%), < 0.6 ng/ml (specificity, 80%), and < 5 pmol/l (specificity, 78%), respectively (**Table 2**). When the concomitant blood glucose level at end-of-fast testing was 2.8–3.3 mmol/l, the specificity of all parameters decreased to only 50%–70% (**Supplementary Table 5**).

## Discussion

This was the first and the largest cohort to propose new diagnostic thresholds for the diagnosis of endogenous hyperinsulinemic hypoglycemia in Chinese populations. This was also one of the largest series of surgically proven insulinoma studies in the literature. We found that when the concomitant blood glucose level at end-of-fast testing was ≤ 2.8 mmol/l, an insulin cutoff value of 5.5 μIU/ml, a C-peptide cutoff value of 0.7 ng/ml, and a proinsulin cutoff value of 12 pmol/l had the best diagnostic accuracy. These new criteria were different from those based on Western populations and yield satisfactory accuracy. We further evaluated the diagnostic performance of the old standards in our cohort, which generated good sensitivity but much poorer specificity.

The most widely used diagnostic criteria for endogenous hyperinsulinemia yielded excellent accuracy in the original studies where these cutoff values were established (4, 7, 8). However, the specificities of these parameters were tested to be significantly lower in other studies, even in the same center, and were less than 80% (11–13), which may have been due to the remarkably small number of cases in the control group in the original studies. Moreover, those criteria have never been tested in non-Western populations. Consistent with previous studies, we also found that these established criteria had high sensitivity but poor specificity in our cohort, suggesting that our study reflected the actual accuracy of these parameters with such cutoff values.

Our newly established cutoff values were higher than the traditional ones, which greatly increased the specificity but did not affect the sensitivity. In previous studies, efforts have also been made to increase the specificity. Vezzosi et al. (11) proposed a higher C-peptide threshold of 0.7 ng/ml and a higher proinsulin threshold of 22 pmol/l, increasing their specificity to 84% and 100% but lowering the sensitivity of both to 76% when the blood glucose level was between 2.5 and 3.3 mmol/l. In the NIH series of studies on insulinoma, the researchers proposed to increase the insulin and proinsulin thresholds to 5 μIU/ml and 22 pmol/L, respectively, leading to higher specificity of 98% and 100% but lowering the sensitivity for insulin to 91%, regardless of the blood glucose level (13).

The specific reasons for the inconsistency of diagnostic criteria among different centers have not been fully clarified. One of the reasons might be attributed to the different concomitant blood glucose levels with which the cut-offs were generated (4, 11, 13). Like in our study, there were some difference in cut-offs among different glucose groups because the composition of patients and controls was a little different. Furthermore, at the end of fast testing, the β-cell polypeptides were positively correlated with glucose in controls but negatively correlated with glucose in patients with insulinoma. Thus, it is easy to infer that with decreased blood glucose levels, these indicators will better distinguish insulinoma from control, which has been reported in previous studies (8, 11, 12). So, it is important to report the diagnostic criteria and accuracy of these parameters with the concomitant blood glucose level. In addition to the different glucose levels, the different study population characteristics(like different race, sex ratio and BMI) and test methods might also be the reasons. Therefore, it is possible that experienced centers with numerous cases will set customized cutoff values to achieve best diagnostic performance in situ.

### Strengths and limitations

Our study has the following strengths: first, this was one of the largest series of surgically proven insulinoma, offering the promise of more reliable results; second, we reported the criteria and their diagnostic performance under different glucose conditions and proposed the best ones which provided more detailed evidence.

However, this study has the following limitations: on the one hand, the long time span of controls included in our study may cause greater heterogeneity between controls, and the number of controls was still relatively small, which might affect the evaluation of specificity; on the other hand, the quality of evidence from retrospective studies may not be as high as that from prospective studies; at last, it will be better to verify our results in another larger Chinese cohort, but the rarity of insulinoma makes this difficult right now, so we hope it can be realized in the near future.

## Conclusions

Our study generated newly tailored and optimized diagnostic criteria for endogenous hyperinsulinemic hypoglycemia in a Chinese population. The new criteria of insulin ≥ 5.5 μIU/ml, C-peptide ≥ 0.7 ng/ml, and proinsulin ≥ 12 pmol/l with end-of-fast glucose ≤ 2.8 mmol/l were different from those based on Western populations and yield satisfactory accuracy.

## Data availability statement

The original contributions presented in the study are included in the article/**Supplementary Material**. Further inquiries can be directed to the corresponding authors.

## Ethics statement

The studies involving human participants were reviewed and approved by Institutional Review Board of PUMCH, Chinese Academy of Medical Sciences. The patients/participants provided their written informed consent to participate in this study.

## Author contributions

JY, YiL, LL, YZ, MQ: acquisition of data, analysis and interpretation of data, drafting the article: FP, LX, WL, QX, HZ, WW, YuL: substantial contributions to conception and design, revising manuscript critically for important intellectual content. All authors contributed to the article and approved the submitted version.

## Acknowledgments

The authors would like to thank the CAMS Innovation Fund for Medical Sciences (CIFMS)(2021-1-I2M-002) and all of the participants in this study.

## Conflict of interest

The authors declare that the research was conducted in the absence of any commercial or financial relationships that could be construed as a potential conflict of interest.

## Publisher's note

All claims expressed in this article are solely those of the authors and do not necessarily represent those of their affiliated organizations, or those of the publisher, the editors and the reviewers. Any product that may be evaluated in this article, or claim that may be made by its manufacturer, is not guaranteed or endorsed by the publisher.
