# Supplementary material for: The optimal diagnostic criteria of endogenous hyperinsulinemic hypoglycemia based on a large cohort of Chinese patients
Source: Front Endocrinol (Lausanne). 2022 Oct 19;13:994707. doi: 10.3389/fendo.2022.994707 (PMC9627162; doi:10.3389/fendo.2022.994707)
Supplement: Supplementary file 1 [file DataSheet_1.docx]

**Supplementary Table 1** Blood glucose distribution pattern at the end of fasting test

| Glucose(mmol/l) | **≤2.0** | **2.0-2.5** | **2.5-2.8** | **2.8-3.0** | **3.0-3.3** | **>3.3** |
| --- | --- | --- | --- | --- | --- | --- |
| control(n) | 0 | 3 | 6 | 3 | 12 | 16 |
| insulinoma(n) | 49 | 65 | 22 | 7 | 1 | 0 |

**Supplementary Table 2** The relationships between blood glucose and insulin, C-peptide and proinsulin at the end of fasting test

|  | Insulin | | C-peptide | | Proinsulin | |
| --- | --- | --- | --- | --- | --- | --- |
|  | r | p | r | p | r | p |
| Insulinoma | -0.415 | <0.001* | -0.378 | <0.001* | -0.151 | 0.149 |
| Controls | 0.369 | 0.019* | 0.462 | 0.004* | 0.385 | 0.048* |

*p＜0.05 was considered significant.

**Supplementary Table 3** Insulin, C-peptide and proinsulin in different blood glucose groups

| Glucose（mmol/l） | ≤2.8 | | ≤3.0 | | ≤3.3 | | 2.8-3.3 | |
| --- | --- | --- | --- | --- | --- | --- | --- | --- |
|  | Controls | Insulinoma | Control | Insulinoma | Control | Insulinoma | Control | Insulinoma |
| Insulin  (μIU/ml） | 1.63  (0.5-5.12) | 24.63  (4.1-185.64)^*^ | 1.49  (0.5-5.12) | 23.27  (4.1-185.64)^*^ | 2.41  (0.5-12.17) | 23.31  (4.1-185.64)^*^ | 2.68  (0.9-12.17) | 17.77  (5.75-40.16)^*^ |
| 1. peptide   (ng/ml) | 0.32  (0.14-0.67) | 2.89  (0.73-14.01)^*^ | 0.33  (0.14-0.82) | 2.83  (0.73-14.01)^*^ | 0.41  (0.14-0.91) | 2.86  (0.73-14.01)^*^ | 0.45  (0.31-0.91) | 2.4  (1.14-3.49)^*^ |
| Proinsulin  (pmol/l) | 2.0  (0.2-11.2) | 79  (2.3-480.7)^*^ | 2.3  (0.2-11.2) | 79  (2.3-480.7)^*^ | 3.1  (0.2-11.8) | 79  (2.3-480.7)^*^ | 4.4  (0.2-11.8) | 61.9  (11.7-230.6)^*^ |

All data were expressed as median（range）

*p＜0.05 was considered significant.

**Supplementary Table 4** Diagnostic performance of new criteria in our study

|  | Patients Meeting Criteria | | Sensitivity %  (95% CI) | Specificity %  (95% CI) | PPV %  (95% CI) | NPV %  (95% CI) |
| --- | --- | --- | --- | --- | --- | --- |
|  | Insulinoma | Control |  |  |  |  |
| **All glucose** |  |  |  |  |  |  |
| Insulin>=5.5μIU/ml | 142/143 | 6/40 | 99.3  (95.6,99.9) | 85  (69.5,93.8) | 95.9  (90.9,98.3) | 97.1  (83.4,99.9) |
| C-peptide>=1.1ng/ml | 136/141 | 3/37 | 96.5  (91.5,98.7) | 91.9  (76.9,97.9) | 97.8  (93.3,99.4) | 87.2  (71.8,95.2) |
| Proinsulin>=12pmol/L | 94/101 | 1/27 | 93.1  (85.8,96.9) | 96.3  (79.1,99.8) | 98.9  (93.4,99.9) | 78.8  (60.6,90.4) |
| **Glucose<=3.3mmol/L** |  |  |  |  |  |  |
| Insulin>=5.5μIU/ml | 142/143 | 1/24 | 99.3  (95.6,99.9) | 95.8  (76.9,99.8) | 99.3  (95.6,99.9) | 95.8  (76.9,99.8) |
| C-peptide>=0.9ng/ml | 140/141 | 1/22 | 99.3  (95.5,99.9) | 95.5  (75.1,99.8) | 99.3  (95.5,99.9) | 95.5  (75.1,99.8) |
| Proinsulin>=12pmol/L | 86/93 | 0/20 | 92.5  (84.6,96.7) | 100  (79.9,100) | 100  (94.7,100) | 74.1  (53.4.88.1) |
| **Glucose<=3.0mmol/L** |  |  |  |  |  |  |
| Insulin>=5.5μIU/ml | 141/142 | 0/12 | 99.3  (95.6,99.9) | 100  (69.9,100) | 100  (96.7,100) | 92.3  (62.1,99.6) |
| C-peptide>=0.9ng/ml | 139/140 | 0/10 | 99.3  (95.5,99.9) | 100  (65.5,100) | 100  (96.7,100) | 90.9  (57.1,99.5) |
| Proinsulin>=12pmol/L | 86/93 | 0/9 | 92.5  (84.6,96.7) | 100  (62.9,100) | 100  (94.7,100) | 56.3  (30.6,79.2) |
| **Glucose<=2.8mmol/L** |  |  |  |  |  |  |
| insulin>=5.5μIU/ml | 134/135 | 0/9 | 99.3  (95.3,100) | 100  (62.9,100) | 100  (96.5,100) | 90  (54.1,99.5) |
| C-peptide>=0.7ng/ml | 133/133 | 0/8 | 100  (96.5,100) | 100  (59.8,100) | 100  (96.5,100) | 100  (59.8,100) |
| Proinsulin>=12pmol/L | 83/89 | 0/8 | 93.2  (85.4,97.2) | 100  (59.8,100) | 100  (94.5,100) | 57.1  (29.6,81.2) |
| **Glucose 2.8-3.3mmol/L** |  |  |  |  |  |  |
| insulin>=5.5μIU/ml | 8/8 | 1/15 | 100  (59.8,100) | 93.3  (66.0,99.7) | 88.8  (50.7,99.4) | 100  (73.2,100) |
| C-peptide>=0.9ng/ml | 8/8 | 1/14 | 100  (59.8,100) | 92.9  (64.2,99.6) | 88.8  (50.6,99.4) | 100  (71.7,100) |
| Proinsulin>=8pmol/L | 4/4 | 1/12 | 100  (39.6,100) | 91.7  (59.8,99.6) | 80.0  (29.8,98.9) | 100  (67.8,100) |

CI, confidence interval; PPV, positive predictive value; NPV, negative predictive value.

**Supplementary Table 5**  Diagnostic performance of western population-based criteria for endogenous hyperinsulinemic hypoglycemia in Chinese people

|  | Patients Meeting Criteria | | Sensitivity %  (95% CI) | Specificity %  (95% CI) | PPV %  (95% CI) | NPV %  (95% CI) |
| --- | --- | --- | --- | --- | --- | --- |
|  | Insulinoma | Control |  |  |  |  |
| **All glucose** |  |  |  |  |  |  |
| insulin>=3.0μIU/ml | 143/143 | 17/40 | 100  (96.7,100) | 57.5  (41.0,72.6) | 89.4  (83.3,93.5) | 100  (82.2,100) |
| C-peptide>=0.6ng/ml | 141/141 | 14/37 | 100  (96.7,100) | 62.2  (62.2,77.1) | 90.9  (85.0,94.8) | 100  (82.2,100) |
| Proinsulin>=5pmol/L | 98/101 | 11/27 | 97.0  (90.9,99.2) | 59.3  (39.0,76.9) | 89.9  (82.3,94.6) | 84.2  (59.5,95.8) |
| **Glucose<=3.3mmol/L** |  |  |  |  |  |  |
| insulin>=3.0μIU/ml | 143/143 | 9/24 | 100  (96.7,100) | 62.5  (40.8,80.4) | 94.1  (88.7,97.1) | 100  (74.7,100) |
| C-peptide>=0.6ng/ml | 141/141 | 5/22 | 100  (96.7,100) | 77.3  (54.2,91.3) | 96.6  (91.8,98.7) | 100  (77.1,100) |
| Proinsulin>=5pmol/L | 90/93 | 8/20 | 96.8  (90.2,99.2) | 60  (36.4,80.0) | 91.8  (84.1,96.2) | 80  (51.4,94.7) |
| **Glucose<=3.0mmol/L** |  |  |  |  |  |  |
| insulin>=3.0μIU/ml | 142/142 | 2/12 | 100  (96.7,100) | 83.3  (50.9,97.1) | 98.6  (94.5,99.8) | 100  (65.5,100) |
| C-peptide>=0.6ng/ml | 140/140 | 2/10 | 100  (96.7,100) | 80  (44.2,96.5) | 98.6  (94.5,99.8) | 100  (59.8,100) |
| Proinsulin>=5pmol/L | 90/93 | 2/9 | 96.9  (90.2,99.2) | 77.8  (40.2,96.1) | 97.8  (91.6,99.6) | 70  (35.4,91.9) |
| **Glucose<=2.8mmol/L** |  |  |  |  |  |  |
| insulin>=3.0μIU/ml | 135/135 | 2/9 | 100  (87.7,100) | 77.8  (40.2,96.1) | 94.6  (94.6,99.1) | 100  (56.1,100) |
| C-peptide>=0.6ng/ml | 132/133 | 1/8 | 99.2  (95.3,99.9) | 87.5  (46.7,99.3) | 99.2  (95.3,99.9) | 87.5  (46.7,99.3) |
| Proinsulin>=5pmol/L | 86/89 | 2/8 | 96.6  (89.7,99.1) | 75  (35.6,95.5) | 97.7  (91.3,99.6) | 66.7  (30.9,90.9) |
| **Glucose 2.8-3.3mmol/L** |  |  |  |  |  |  |
| insulin>=3.0μIU/ml | 8/8 | 8/15 | 100  (59.8,100) | 46.7  (22.3,72.6) | 50  (25.5,74.5) | 100  (56.1,100) |
| C-peptide>=0.6ng/ml | 8/8 | 4/14 | 100  (59.8,100) | 71.4  (42.0,90.4) | 66.7  (35.4,88.7) | 100  (65.5,100) |
| Proinsulin>=5pmol/L | 4/4 | 6/12 | 100  (39.6,100) | 50  (22.3,77.7) | 40  (13.7,72.6) | 100  (51.7,100) |

CI, confidence interval; PPV, positive predictive value; NPV, negative predictive value.


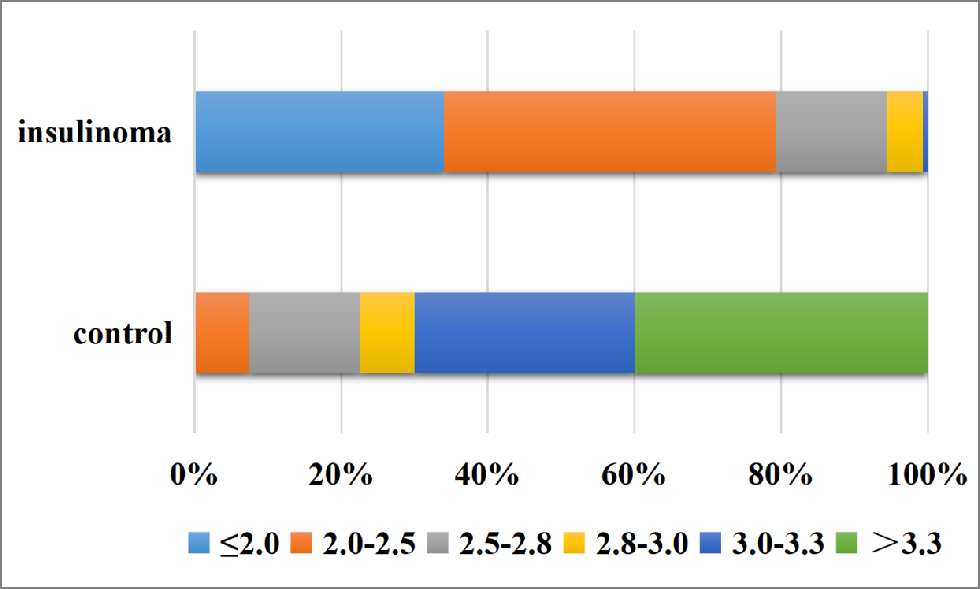


**Supplementary Figure 1 Blood glucose concentrations at the end of the fasting test.** The above column represents the blood glucose distribution at the end of the fasting test in patients with insulinoma; the below column represents the blood glucose distribution at the end of the fasting test in controls.
